# Preparation and Electrocatalytic Hydrogen Evolution Performance of CoS_2_:Mo Microrods

**DOI:** 10.3390/molecules31071131

**Published:** 2026-03-30

**Authors:** Shuai Shao, Xiaocan Liu, Ping Liang, Weiye Yang, Lijian Meng, Hongyan Peng, Shihua Zhao

**Affiliations:** 1 College of Physics and Electronic Engineering, Hainan Normal University, Haikou 571158, China; 202412070200011@hainnu.edu.cn (S.S.); 13637696108@163.com (X.L.); 202212070200007@hainnu.edu.cn (P.L.); haha_weiye@163.com (W.Y.); mdjphy@163.com (H.P.); 2 CIETI/ISEP, Polytechnic of Porto, Rua Dr. António Bernardino de Almeida, 4249-015 Porto, Portugal; ljm@isep.ipp.pt; 3 The Innovation Platform for Academicians of Hainan Province, Haikou 571158, China

**Keywords:** hydrogen evolution reaction, cobalt disulfide, doping, electrochemistry, molybdenum

## Abstract

Cobalt disulfide (CoS_2_) features highly active catalytic sites and is regarded as a promising candidate for electrocatalytic hydrogen evolution. In this study, molybdenum-doped cobalt disulfide (CoS_2_:Mo) was synthesized via a facile hydrothermal approach. XRD analysis confirms that the obtained samples crystallize in a cubic pyrite structure, with diffraction peaks consistently shifting towards lower angles. SEM characterization reveals that the samples exhibit microrod-like morphologies with an average size of approximately 1 μm. Integrated analyses from XRD, XPS, and EDS mapping demonstrate that Mo is uniformly distributed across the surface and successfully doped into the CoS_2_ lattice. Electrochemical measurements indicate that the CoS_2_:Mo sample delivers a low overpotential of 122 mV and a Tafel slope of 128 mV dec^−1^ at a current density of 10 mA cm^−2^ in alkaline media, significantly surpassing the performance of pure CoS_2_ and MoS_2_. Moreover, the CoS_2_:Mo exhibits an enhanced double-layer capacitance, with a C_dl_ value of 2.72 mF cm^−2^, superior to that of pure CoS_2_ (1.63 mF cm^−2^) and MoS_2_ (0.31 mF cm^−2^). Mo doping enhances conductivity and active sites, thereby boosting electrocatalysis. This work presents an effective strategy for the development of cost-efficient and high-performance non-precious metal electrocatalysts.

## 1. Introduction

Amid the dual challenges posed by the global energy crisis and environmental pollution, the advancement of efficient hydrogen production technologies has garnered widespread attention from both the scientific and industrial communities, as hydrogen is recognized as a clean and renewable energy carrier capable of replacing conventional fossil fuels [1,2,3,4]. Currently, there are numerous methods for hydrogen production, such as conventional hydrogen generation from fossil fuels, hydrogen production using modern, inexpensive, and non-toxic photocatalysts [5,6,7,8], and hydrogen release via the decomposition of hydrogen carriers [9,10,11]. Among these technologies, the hydrogen evolution reaction (HER) via water electrolysis is considered a highly promising approach for the future hydrogen economy due to its environmentally benign nature. Nevertheless, the commercialization of water electrolysis remains hindered by the lack of catalytic materials that are simultaneously efficient, stable, and cost-effective. As such, the development of high-performance electrocatalysts is at the core of this technology [12,13,14,15,16,17,18]. The HER process typically requires an additional overpotential to overcome sluggish water dissociation kinetics and thermodynamic limitations [19,20,21,22]. Therefore, electrocatalysts capable of facilitating high reaction rates and achieving elevated current densities at low overpotentials are essential [23,24,25]. Currently, platinum-based catalysts exhibit the highest performance; however, their large-scale application is restricted by limited availability and high cost [26,27,28,29]. To address this, considerable efforts have been directed toward exploring non-precious metal-based alternatives, including transition metal sulfides [30,31,32,33,34], selenides [35,36,37], phosphides [38,39,40], and carbides [41,42]. Among these non-precious metal HER catalysts [43,44,45], pyrite (CoS_2_) has emerged as a prominent candidate owing to its low cost, environmental compatibility, and abundance of active sites [46,47].

Despite its advantages, pure CoS_2_ still exhibits limitations in catalytic performance due to its relatively wide band gap, inadequate electrical conductivity, and suboptimal adsorption energy for reaction intermediates. To address these issues and enhance the HER activity of cobalt disulfide, various modification strategies have been explored. For example, Liu et al. [48] fabricated a hybrid heterojunction electrode with dual interfaces (MoS_2_/CoSAs-NS-CNTs@CoS_2_/CC) on carbon cloth, enabling vectorial electron transport pathways to improve electrocatalytic efficiency. Myung-In Kim et al. [49] synthesized a heterostructure comprising 1T/2H-phase MoS_2_ and CoS_2_ on nitrogen-doped carbon nanofibers (1T/2H-MoS_2_/CoS_2_@NCNF) via electrospinning and hydrothermal methods. Chen et al. [50] embedded CoS_2_ into nitrogen-doped carbon nanotubes (CoS_2_/NCNHP), thereby increasing the specific surface area and enhancing electrical conductivity. Although these methods substantially improved HER performance, they generally involved complex synthesis procedures and relatively high costs. To simplify the process, Sankar Sekar et al. [51] prepared an rGO-CoS_2_ nanocomposite using a facile one-pot hydrothermal approach. The resulting material exhibited an overpotential of 377 mV and a Tafel slope of 121 mV·dec^−1^ at 10 mA·cm^−2^ in 1 M KOH electrolyte. He et al. [52] developed Sn-doped CoS_2_ nanowire arrays (Sn-CoS_2_/CC) on carbon cloth, achieving an overpotential of 161 mV at 10 mA·cm^−2^ in 1 M KOH, thereby simplifying the preparation process while still offering room for performance enhancement.

In this study, Mo-doped CoS_2_ (CoS_2_:Mo) was synthesized via a simple and cost-effective hydrothermal method, Mo doping not only enhances water molecule adsorption capacity and significantly boosts catalytic activity, but also improves the stability of sulfides [53,54], significantly reducing synthesis complexity while improving HER catalytic activity. The resulting catalyst demonstrates a low overpotential of 122 mV and a Tafel slope of 128 mV·dec^−1^ at a current density of 10 mA·cm^−2^ in 1.0 M KOH, indicating a notable enhancement in hydrogen evolution electrocatalytic performance.

## 2. Results

### 2.1. Analysis of Structure and Composition via XRD

**Figure 1:**
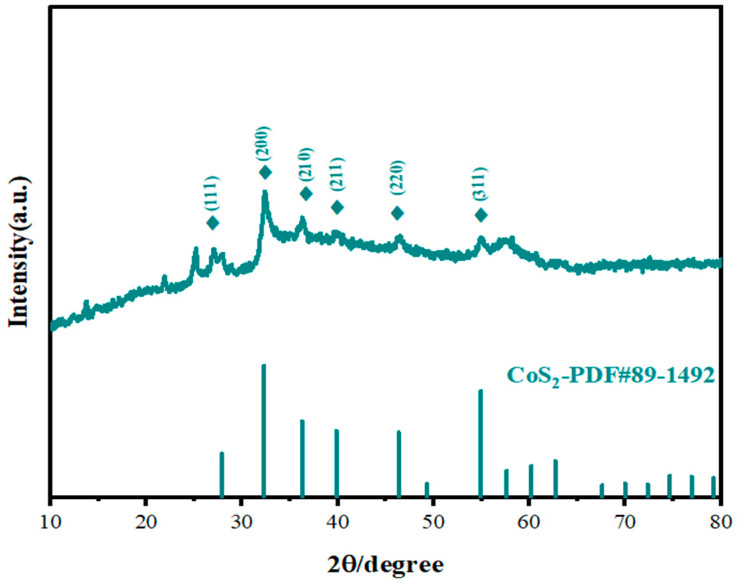
XRD pattern of CoS_2_:Mo.

Figure 1 displays the XRD pattern of the CoS_2_:Mo sample. The diffraction peaks located at 27.1°, 32.3°, 36.2°, 39.9°, 46.3°, and 55.1° correspond to the (111), (200), (210), (211), (220), and (311) planes of CoS_2_ (PDF#89-1492), respectively. These results confirm the successful formation of crystalline CoS_2_ following gas-phase sulfidation at 450 °C. Compared with the standard CoS_2_ pattern, a slight shift in diffraction peaks toward lower angles is observed, indicating lattice expansion due to Mo incorporation. This shift is attributed to the larger atomic radius of Mo relative to Co [55]. Notably, no diffraction peaks associated with MoS_2_ are detected, suggesting that Mo doping does not introduce any secondary crystalline phase.

### 2.2. Raman Analysis

**Figure 2:**
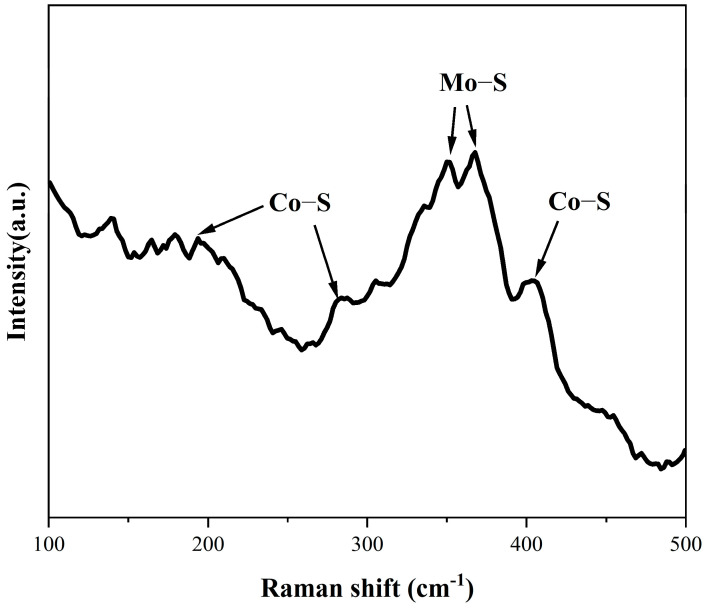
Raman pattern of CoS_2_:Mo.

To further analyze the crystal structure, Raman spectroscopy was conducted (Figure 2). The characteristic Raman bands near 282 cm^−1^ and 400 cm^−1^ are assigned to the E_9_ and A_9_ vibrational modes of Co-S bonds, respectively [56]. A peak observed at approximately 190 cm^−1^ is also attributed to CoS_2_ vibrations [57]. In addition, peaks near 349 cm^−1^ and 367 cm^−1^ are associated with Mo-S vibrations [41,57], supporting the incorporation of Mo into the CoS_2_ matrix.

### 2.3. Elemental Analysis of the Catalysts by XPS

**Figure 3:**
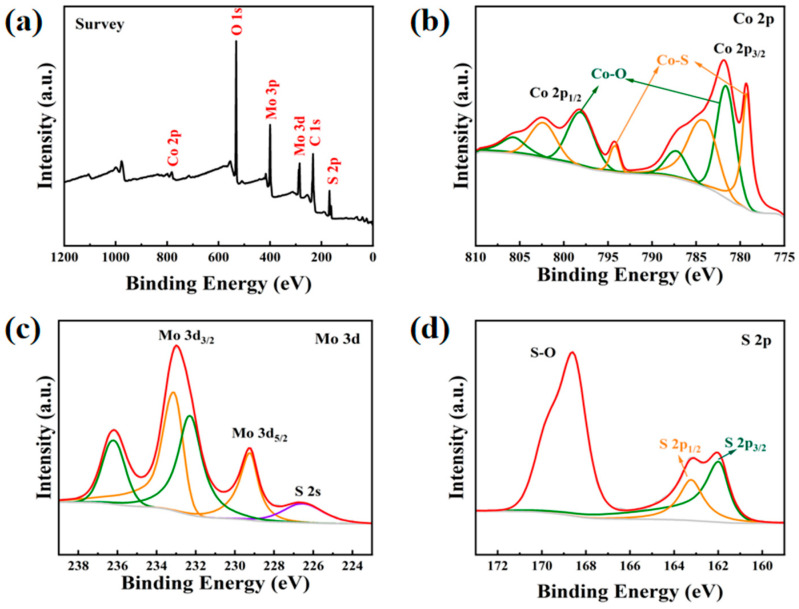
Elemental analysis of the catalysts. (**a**) XPS survey spectra and high-resolution spectra of (**b**) Co 2p (**c**) Mo 3d, and (**d**) S 2p of CoS_2_:Mo.

XPS was employed to investigate the elemental composition and valence states of CoS_2_:Mo. As shown in Figure 3, the full survey spectrum (Figure 3a) reveals the presence of C, O, S, Co, and Mo. The Mo signal at approximately 400 eV confirms successful Mo incorporation, while the O signal originates from surface-adsorbed oxygen and oxidation during air exposure [41]. The high-resolution Co 2p spectrum (Figure 3b) exhibits peaks at 781.6 eV and 798.2 eV, along with their satellite peaks, which are attributed to Co-O bonding [58]. The Mo 3d spectrum (Figure 3c) shows peaks at 229.3 eV and 233.1 eV, corresponding to Mo^4+^ 3d_5_/_2_ and Mo^4+^ 3d_3_/_2_, respectively. Additional peaks at 232.3 eV and 236.2 eV are assigned to Mo^6+^, indicating the coexistence of Mo^4+^ and Mo^6+^ oxidation states, with Mo^4+^as the dominant species. The presence of Mo^6+^ is ascribed to surface oxidation upon air exposure [58]. A characteristic peak at 226.6 eV corresponds to the S 2s orbital. The S 2p spectrum (Figure 3d) shows peaks at 162.0 eV and 163.2 eV, attributed to S 2p_3_/_2_ and S 2p_1_/_2_, respectively, confirming the formation of metal–sulfide bonds. Peaks at 168.5 eV and 169.7 eV are associated with S-O bonds, further indicating surface oxidation [58,59].

### 2.4. Morphological Characterization

**Figure 4:**
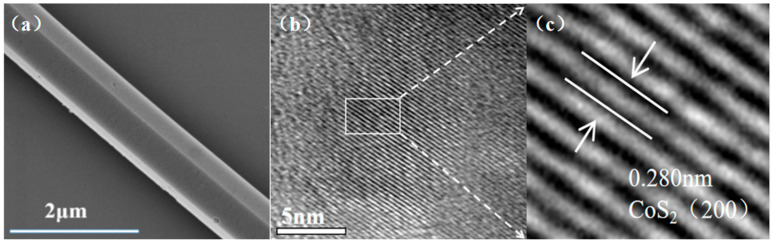
(**a**) SEM and (**b**,**c**) HRTEM images of CoS_2_:Mo.

Figure 4 presents the SEM and TEM images of the CoS_2_:Mo sample. As illustrated in Figure 4, the sample exhibits a rod-like morphology with a typical length of approximately 1 μm. The high-resolution TEM image in Figure 4 reveals well-defined lattice fringes, with an interplanar spacing of approximately 0.280 nm, corresponding to the (200) plane of CoS_2_. This observation is consistent with the XRD results.

### 2.5. EDS Analysis

**Figure 5:**
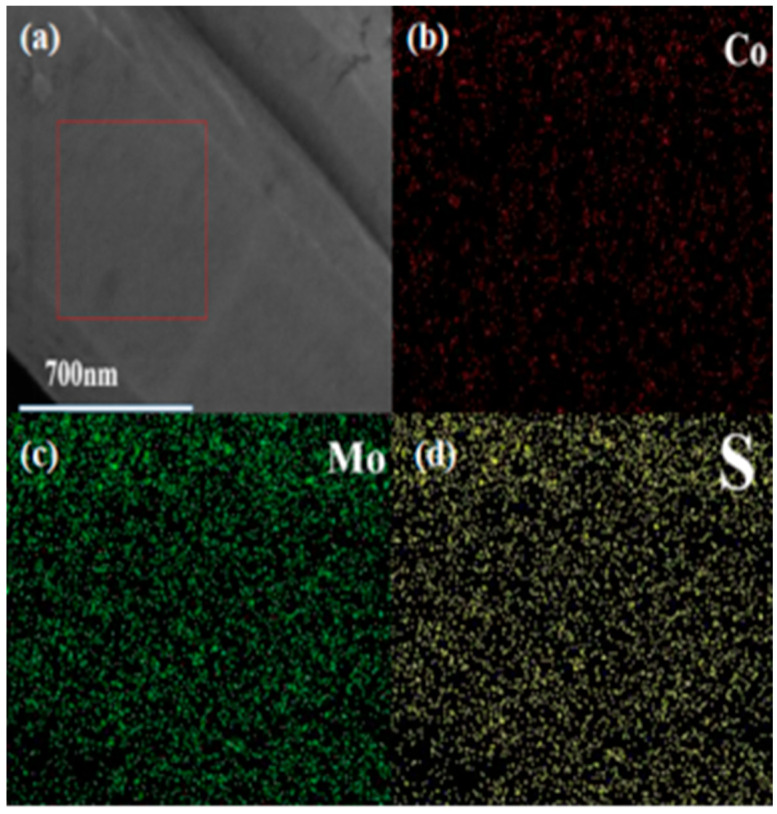
EDS mapping images of CoS_2_:Mo. (**a**) The selected location. The elemental distribution map of (**b**) Co, (**c**) Mo and (**d**) S.

Figure 5a–d show the EDS elemental mapping images of CoS_2_:Mo. The results confirm the uniform distribution of Co, Mo, and S throughout the sample, indicating successful and homogeneous Mo doping within the CoS_2_ lattice.

## 3. Discussion

### 3.1. Electrochemical Measurements in Alkaline Electrolytes

**Figure 6:**
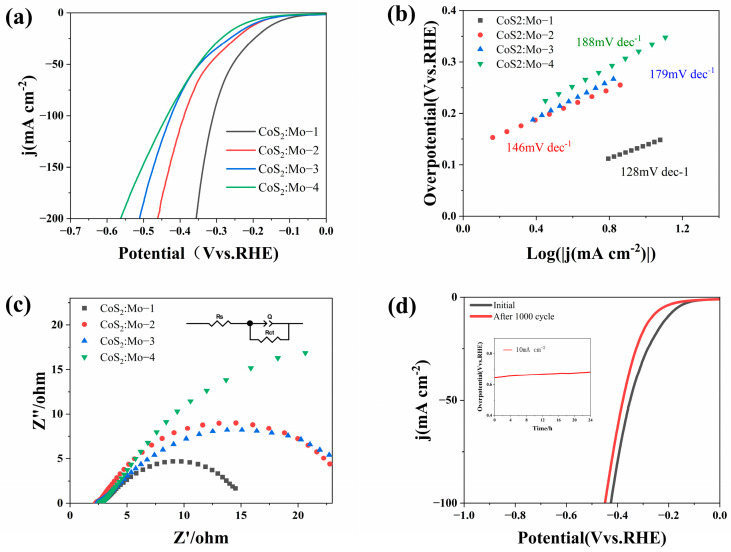
Electrochemical performance of the catalysts toward the HER in 1 M KOH. (**a**) Linear sweep voltammetry curves of CoS_2_:Mo-1, CoS_2_:Mo-2, CoS_2_:Mo-3, and CoS_2_:Mo-4. (**b**) Tafel slopes and (**c**) EIS spectra. (**d**) Linear sweep voltammetry curves of CoS_2_:Mo-1 before and after 1000 CV cycles, with the inset showing the chronopotentiometry curve of CoS_2_:Mo-1 tested at a current density of 10 mA cm^−2^ for 24 h.

To evaluate the electrochemical performance of the synthesized materials, a series of tests were conducted. For comparison, MoS_2_ and CoS_2_ were synthesized using the same hydrothermal method. The HER performance of the catalysts was assessed in an alkaline medium (1 mol/L KOH) using a standard three-electrode system with 90% iR compensation. Figure 6a presents the linear sweep voltammetry (LSV) curves of samples with different Co:Mo doping ratios. As shown in Figure 6a, at a current density of 10 mA cm^−2^, CoS_2_:Mo-1 exhibits the lowest overpotential (η_10_ = 122 mV), which is significantly lower than those of CoS_2_:Mo-2, CoS_2_:Mo-3, and CoS_2_:Mo-4, indicating that the sample with a Co:Mo doping ratio of 1:1 achieves the best hydrogen evolution performance. Figure 6b shows the Tafel slopes of the samples. It can be seen from Figure 6b that CoS_2_:Mo-1 has the smallest Tafel slope, implying that under the same applied potential, CoS_2_:Mo-1 exhibits the fastest increase in current density. Figure 6c displays the electrochemical impedance spectroscopy (EIS) Nyquist plots. As evident from Figure 6c, CoS_2_:Mo-1 possesses the smallest semicircle diameter, indicating the lowest charge transfer resistance and thus the highest electrical conductivity among all samples. These results indicate that the electrochemical performance of the sample is enhanced as the doping ratio increases. Furthermore, the stability of the CoS_2_:Mo-1 sample was evaluated. Figure 6d presents the LSV curves before and after 1000 cycles of cyclic voltammetry testing, with the inset showing the chronoamperometry curve recorded over 24 h at a constant current density of 10 mA cm^−2^. The LSV curves exhibit negligible change after 1000 cycles, and the operating potential remains stable throughout the 24 h test, confirming the excellent long-term stability of the CoS_2_:Mo-1 catalyst.

**Figure 7:**
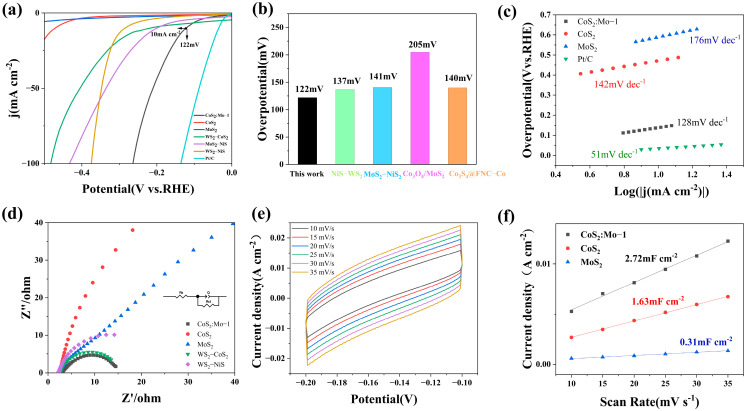
Electrochemical performance of the catalysts toward the HER in 1 M KOH. (**a**) Linear sweep voltammetry (LSV) curves of Pt/C, CoS_2_:Mo, MoS_2_, CoS_2_, and other sulfur-based catalysts for hydrothermal synthesis. (**b**) Comparison of HER performance with recently reported TMDs. (**c**) Tafel slope and (**d**) EIS curves for different catalysts (inset: equivalent circuit diagram). (**e**) CV curves of CoS_2_:Mo at different scan rates. (**f**) C_dl_ for different catalysts.

Figure 7a presents the linear sweep voltammetry (LSV) curves of the samples. As shown in Figure 7a, the CoS_2_:Mo material exhibits a low overpotential of 122 mV at a current density of 10 mA cm^−2^ in 1 M KOH. Although this is higher than the overpotential of 36 mV for commercial Pt/C, it is significantly lower than those of MoS_2_ and CoS_2_. Additionally, its performance surpasses that of WS_2_-CoS_2_ (η_10_ = 218 mV), MoS_2_-NiS (η_10_ = 199 mV), and WS_2_-NiS (η_10_ = 285 mV), which were also prepared using the same hydrothermal method. Furthermore, the CoS_2_:Mo sample was compared with other non-precious metal catalysts. Figure 7b shows a comparison with various non-precious metal catalysts, demonstrating that the CoS_2_:Mo sample outperforms them under identical conditions [59,60,61,62]. These results clearly indicate that Mo doping significantly enhances the alkaline HER performance of CoS_2_ catalysts. Table 1 compares the overpotential of the catalyst developed in this work with other reported Co-, S-, and Mo-based catalysts. As shown in Table 1, our catalyst exhibits the lowest overpotential at a current density of 10 mA cm^−2^, indicating its superior hydrogen evolution performance.

The dynamic process associated with the Tafel slope also provides insights into the enhanced HER performance. The Tafel slope was determined using the following equation

η = b log (j) + a(1)

where b is the Tafel slope and j is the current density, to investigate the hydrogen evolution reaction kinetics. Figure 7c presents the Tafel curves of the samples. As shown in Figure 7c, except for Pt/C (51 mV·dec^−1^), the Mo:CoS_2_ sample exhibits the smallest Tafel slope of 128 mV·dec^−1^, which is lower than those of MoS_2_ (176 mV·dec^−1^) and CoS_2_ (142 mV·dec^−1^). This indicates that the CoS_2_:Mo material follows the Volmer mechanism [63]. Around a current density of 10 mA·cm^−2^, the current density of CoS_2_:Mo exhibits the most rapid change with respect to the overpotential. A lower Tafel slope corresponds to a faster increase in current, significantly enhancing the electrocatalytic hydrogen evolution reaction rate. These findings further demonstrate that CoS_2_:Mo possesses the fastest HER kinetics and the best HER catalytic performance among the prepared materials. Moreover, Mo doping improves the Tafel slope of the material, suggesting enhanced charge transfer efficiency and accelerated reaction kinetics following Mo incorporation, which results in the most pronounced catalytic reaction kinetics under the same mechanism.

Electrochemical impedance spectroscopy (EIS) measurements were employed to assess the electrocatalytic kinetics at the electrolyte–electrocatalyst interface [64,65,66,67,68,69,70]. Figure 7d presents the electrochemical impedance spectra of the samples. The smaller charge transfer resistance, as indicated by the smallest semicircle diameter for this catalyst, suggests faster electron transfer kinetics following doping. The significant enhancement in conductivity demonstrates that Mo doping effectively reduces charge transfer resistance, promotes electron transport, and thereby optimizes catalytic activity.

The electrochemical surface area (ECSA) of the electrocatalysts was evaluated via electrochemical double-layer capacitance (C_dl_) measurements. The C_dl_ values were derived from CV tests conducted at varying scan rates. Figure 7e,f display the cyclic voltammetry curves and the corresponding fitted double-layer capacitance curves for the samples, respectively. The results reveal that the Mo-doped sample exhibits a C_dl_ value of 2.72 mF·cm^−2^, which is higher than those of MoS_2_ (0.31 mF·cm^−2^) and CoS_2_ (1.63 mF·cm^−2^). This suggests that Mo doping effectively enhances the electrochemical active surface area, thereby exposing more HER active sites, which is in agreement with the improved HER catalytic activity shown in Figure 7a.

### 3.2. Electrochemical Measurements in Acidic Electrolytes

**Figure 8:**
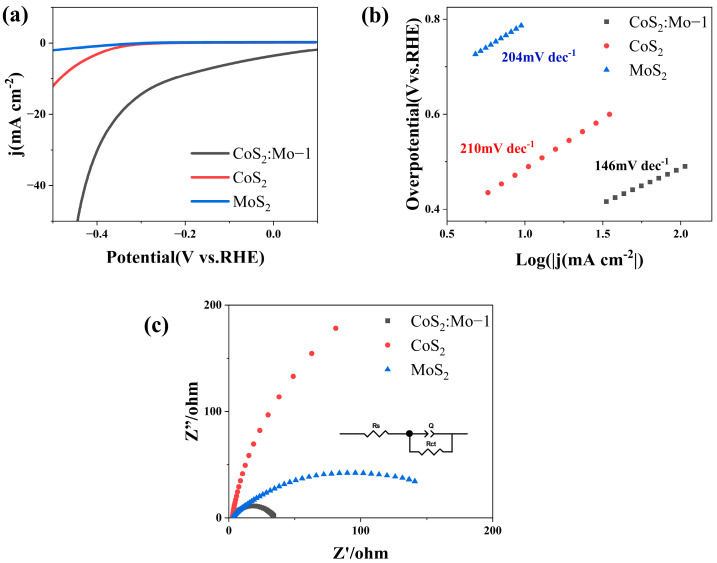
Electrochemical performance of the catalysts toward the HER in 0.5 M H_2_SO_4_. (**a**) LSV curves. (**b**) Tafel slope and (**c**) EIS curves for different catalysts (inset: equivalent circuit diagram).

The HER performance of the CoS_2_:Mo sample and reference catalysts was further evaluated in an acidic electrolyte (0.5 mol/L H_2_SO_4_). As shown in Figure 8a, the linear sweep voltammetry (LSV) curves reveal that CoS_2_:Mo exhibits superior electrocatalytic activity, achieving a low overpotential of 227 mV at a current density of 10 mA cm^−2^, outperforming both MoS_2_ and CoS_2_. The linear regions of the LSV curves were fitted using the Tafel equation to derive the corresponding Tafel slopes. Figure 8b presents the resulting Tafel plots, indicating that CoS_2_:Mo displays a Tafel slope of 146 mV·dec^−1^, which is substantially lower than those of MoS_2_ (204 mV·dec^−1^) and CoS_2_ (210 mV·dec^−1^), suggesting enhanced catalytic kinetics.

The interfacial charge transfer kinetics were investigated using EIS. Figure 8c presents the electrochemical impedance spectra of the samples under acidic conditions. All three curves exhibit similar profiles, characterized by semicircles in the high-frequency region. Notably, CoS_2_:Mo presents the smallest semicircle diameter, indicating the lowest charge transfer resistance, which reflects faster HER kinetics at the electrocatalyst–electrolyte interface. The improved HER activity of CoS_2_:Mo is primarily attributed to the enhanced electrical conductivity induced by Mo incorporation.

## 4. Materials and Methods

### 4.1. Experimental Reagents

Cobalt nitrate hexahydrate (Co(NO_3_)_2_ ·6H_2_O, 99%, Macklin, Shanghai, China), ammonium molybdate tetrahydrate ((NH_4_)_6_Mo_7_O_24_·4H_2_O, AR, Sinopharm Chemical Reagent, Shanghai, China), thiourea (99%, Aladdin, Shanghai, China), hydrochloric acid (HCL, Xilong Scientific, Shenzhen, China), anhydrous ethanol (C_2_H_5_OH, Xihua, Shenzhen, China), platinum-on-carbon catalyst (Pt/C, 20% Pt, Macklin, Shanghai, China), carbon cloth, and Nafion solution (5%, DuPont, Wilmington, DE, USA) were used as received without further purification.

### 4.2. Synthesis of CoS_2_:Mo-Doped Materials

Under continuous stirring, a mixture of 2.5 mmol of Co(NO_3_)_2_·6H_2_O and varying amounts (0.35 mmol, 0.18 mmol, 0.07 mmol, and 0.035 mmol) of (NH_4_)_6_Mo_7_O_24_·4H_2_O was gradually dissolved in 30 mL of deionized water. After 10 min of stirring, the resulting solution was transferred into a 50 mL Teflon-lined stainless-steel autoclave and maintained at 150 °C for 6 h.

Upon completion of the reaction, the resulting precipitate was collected and repeatedly washed with deionized water and ethanol to remove impurities. The washed precursor was then dried in an oven at 60 °C for 12 h. Subsequently, a defined amount of the dried precursor was thoroughly mixed and ground with thiourea powder, followed by thermal treatment in a tube furnace. Under an N_2_ atmosphere, the temperature was increased to 450 °C at a heating rate of 5 °C/min and held for 120 min. After calcination, CoS_2_:Mo was obtained. Samples were designated as CoS_2_:Mo-1, CoS_2_:Mo-2, CoS_2_:Mo-3, and CoS_2_:Mo-4 based on the varying amounts of ammonium molybdate used.

### 4.3. Materials Characterization

The morphology and microstructure of the synthesized materials were characterized by scanning electron microscopy (SEM, HITACHI SU8010, 1.0 nm at15 kV, Hitachi, Tokyo, Japan) and transmission electron microscopy (TEM, FEI Tecnai G2 F20, 200 kV, FEI Company, Hillsboro, OR, USA). The crystal phase structure and chemical composition were analyzed using X-ray diffraction (XRD, Ultima IV, Rigaku Holdings Corporation, Tokyo, Japan) and X-ray photoelectron spectroscopy (XPS, K-Alpha, Thermo Fisher Scientific Inc., Waltham, MA, USA).

### 4.4. Electrochemical Measurements

The electrochemical performance was evaluated using a CHI660 (CH Instruments, Shanghai, China) electrochemical workstation in a standard three-electrode configuration, consisting of a platinum sheet as the counter electrode, a reference electrode, and a working electrode. A saturated Ag/AgCl electrode was used as the reference in 0.5 M H_2_SO_4_ electrolyte, while a Hg/HgO electrode served as the reference in 1.0 M KOH solution. The catalyst ink was prepared by ultrasonically dispersing 5 mg of catalyst in a mixture of 150 μL ethanol, 350 μL deionized water, and 10 μL Nafion solution (0.5 wt%). Subsequently, 0.1 mL of the resulting suspension was drop-cast onto a carbon cloth (CF) electrode and dried at room temperature. All measured potentials were converted to the reversible hydrogen electrode (RHE) scale using the following equations:

E(RHE) = E(Hg/HgO) + 0.098 V + 0.059 × pH (pH = 14)(2)

E(RHE) = E(Ag/AgCl) + 0.0592 × pH + 0.197 (pH = 0),(3)

Linear sweep voltammetry (LSV) was performed at a scan rate of 1 mV s^−1^ with 90% iR compensation. Cyclic voltammetry (CV) was conducted in the potential range of 0 to 0.2 V (vs. RHE) at scan rates from 10 to 35 mV s^−1^. Electrochemical impedance spectroscopy (EIS) measurements were carried out over a frequency range of 10^5^ to 0.1 Hz with an AC amplitude of 5 mV. The double-layer capacitance (C_dl_) was calculated from the CV curves recorded at various scan rates (10–35 mV/s) within the non-faradaic potential window of 0–0.2 V (vs. RHE). The electrochemically active surface area (ECSA) was estimated using the following equation:

ECSA = C_dl_/Cs, (4)

## 5. Conclusions

In this study, Mo-doped CoS_2_ (CoS_2_:Mo) microrods were successfully synthesized via a simple hydrothermal method, and their electrocatalytic hydrogen evolution reaction (HER) performance was systematically evaluated. Structural characterization confirms the successful incorporation of Mo into the CoS_2_ crystal lattice and the resulting lattice expansion. The material exhibits a uniform microrod morphology with homogeneous elemental distribution and no detectable impurity phases. Electrochemical tests show that under alkaline conditions (1.0 M KOH), CoS_2_:Mo requires an overpotential of only 122 mV to achieve a current density of 10 mA cm^−2^, significantly outperforming undoped CoS_2_ and MoS_2_. Tafel slope analysis further indicates improved reaction kinetics. Additionally, cyclic stability tests and chronopotentiometry measurements demonstrate good stability of the sample. Moreover, the material also exhibits favorable HER activity in acidic media. The performance enhancement is mainly attributed to the improved electrical conductivity, enlarged electrochemical active surface area, optimized charge transfer, and increased exposure of catalytic active sites resulting from Mo doping. This work not only presents an efficient non-precious metal HER electrocatalyst but also provides a feasible strategy for designing low-cost, high-performance HER catalysts through elemental doping. In future work, in situ characterization and theoretical calculations will be carried out to gain deeper insights into the dynamic structural evolution and reaction mechanisms at the doping sites during HER. Furthermore, the application of the material in overall water splitting or its integration with other renewable energy systems (such as photovoltaics-coupled electrolysis) will be investigated to evaluate its practical potential and facilitate its transition from laboratory research to industrial applications.

## Figures and Tables

**Table 1:** Comparison with Other Co-, S-, and Mo-Based Catalysts.

Catalyst	Preparation Strategy	Electrolyte	Potential (mV)	References
rGO-CoS_2_	hydrothermal	1 mol L^−1^ KOH	377	[51]
Sn-CoS_2_/CC	hydrothermal	1 mol L^−1^ KOH	161	[52]
MoS_2_-NiS_2_	solid-state synthesis	1 mol L^−1^ KOH	141	[60]
Co_3_O_4_/MoS_2_	hydrothermal	1 mol L^−1^ KOH	205	[61]
Co_3_S_4_@FNC-Co	hydrothermal	1 mol L^−1^ KOH	140	[62]
CoS_2_:Mo	hydrothermal	1 mol L^−1^ KOH	122	This work

## Data Availability

The original contributions presented in this study are included in the article. Further inquiries can be directed to the cor-responding author.
